# Cultural adaptation and validation of the mental illness associated stigma scale for Arabic-speaking population in Saudi Arabia

**DOI:** 10.3389/fpsyt.2023.1265096

**Published:** 2024-01-16

**Authors:** Nasser F. BinDhim, Nora A. Althumiri, Sulaiman M. Al-Luhaidan, Mohammed Alhajji, Sami Yahya A. Saad, Hussain Alyami, Anton Svendrovski, Rashed Abdullah Al-Duraihem, Abdulhameed Abdullah Alhabeeb

**Affiliations:** ^1^Informed Decision-Making Research and Studies, Riyadh, Saudi Arabia; ^2^Studies and Information, National Committee for Narcotics Control, Riyadh, Saudi Arabia; ^3^Office of Assistant Minister, Behavioral Insights Unit, Ministry of Health, Riyadh, Saudi Arabia; ^4^Science Department, Alfaisal University, Riyadh, Saudi Arabia; ^5^Department of Neuroscience, King Abdullah Medical City, Mecca, Saudi Arabia; ^6^Department of Internal Medicine, Taif University, Taif, Saudi Arabia; ^7^UZIK Consulting Inc., Toronto, ON, Canada; ^8^National Centre for Mental Health Promotion, Riyadh, Saudi Arabia

**Keywords:** mental illness, stigma, validation, MIAS scale, Arabic, Saudi Arabia, cultural adaptation

## Abstract

**Objective:**

This research aimed to culturally adapt and validate the MIAS scale for Arabic-speaking individuals within the Saudi Arabian general population, with an emphasis on cultural, societal, and individual nuances.

**Methods:**

An initial pilot testing with a small group ensured the scale's clarity. Subsequently, two cross-sectional studies involving 189 participants to assess structural validity of the Arabic MIAS scale, and 38 participants to assess the test-retest reliability. Descriptive statistics, Cronbach's α, Intraclass Correlation Coefficient (ICC), and Confirmatory Factor Analysis (CFA) were employed for data analysis.

**Results:**

The Arabic MIAS scale demonstrated good internal consistency and acceptable test-retest reliability (ICC α = 0.631). A three-factor model emerged (CFI = 0.890, TLI = 0.845, RMSEA = 0.094), including “Outcomes,” “Negative Stereotypes,” and “Recovery,” closely mirroring the original study's structure. one item was excluded from the model since it didn't align with any of the three factors.

**Conclusion:**

The study contributes a culturally adapted, validated, non-condition-specific tool to gauge public attitudes toward mental health stigma in an Arabic context. It highlights the need for culturally sensitive stigma research and interventions and underscores the importance of improving such tools for cross-cultural applicability and comparability.

## Background

Mental health stigma refers to the negative attitudes, misconceptions, and stereotypes that individuals and society hold toward individuals with mental illnesses (1). This stigma can manifest in several ways, including social exclusion, discrimination, and prejudice (2). Individuals suffering from mental health disorders often feel marginalized, misunderstood, and feared by society due to such stigma (3).

Measuring mental health stigma in the general population is crucial to understand the extent and prevalence of these negative attitudes and misconceptions (4). It offers a comprehensive overview that aids in identifying the factors contributing to the stigma and provides data to develop effective public health strategies to combat it (5). Furthermore, it allows policymakers to address stigma within the broader social context and promotes inclusive health services (6).

Measuring mental health stigma in the general population helps to improve the mental health outcomes of individuals suffering from mental disorders. It supports the development of stigma reduction interventions and promotes public understanding and empathy (7). Moreover, it assists in identifying the social and cultural factors that perpetuate stigma, providing valuable insights for tailoring mental health advocacy and education programs (8).

Condition-specific tools for measuring mental health stigma focus on the stigma associated with specific mental health conditions, such as schizophrenia or depression (9). They provide detailed insights into the unique stigma experiences related to individual disorders (10). On the other hand, assessments of public attitudes toward mental health stigma provide a broad understanding of societal attitudes and beliefs about mental illnesses (11). These assessments measure the general public's knowledge, beliefs, and attitudes toward mental health, which can inform public health initiatives and policies (12). While both are essential, condition-specific tools offer a more nuanced understanding of stigma, and assessments of public attitudes provide an overview of societal attitudes toward mental health (13).

However, accurately measuring mental health stigma in the general population poses a significant challenge. This is primarily because stigma is a complex, multidimensional construct, influenced by various factors such as culture, personal beliefs, experiences, and societal norms (3). Thus, a validated scale adapted to the local community is essential for accurately measuring mental health stigma. This entails considering the specific cultural, societal, and individual factors that influence stigma in the community. The process of cultural adaptation ensures that the scale is relevant and sensitive to the local context, thus enhancing its validity (14).

Saudi Arabia, under the guidance of the National Center for Mental Health Promotion (NCMH), has established several programs to monitor mental health indicators at the national level. These include the Saudi Mental Health Surveillance system and an ongoing project aimed at measuring national mental health literacy (15, 16). Additionally, the NCMH is planning to monitor the stigma associated with mental illness within the Saudi general population, which is directly related to the research project presented in this article.

This study aims to adapt and validate the Generic Scale for Public Health Surveillance of Mental Illness Associated Stigma (MIAS) (17) for Arabic-speaking individuals within the general population of Saudi Arabia. This includes translation, assessing the psychometric properties, establishing reliability, and conducting a test-retest reliability.

## Methods

### Selection of a stigma instrument

The study criteria for selecting a tool were based on the need to choose an instrument that measures attitudes toward mental illnesses. We prioritized tools that are concise and have been used nationally, ensuring their validation for international comparisons.

### Design

This study entailed translating the original English version of the MIAS into a culturally and linguistically suitable Arabic version specific to Saudi Arabia. This adaptation was then subjected to a validation study, utilizing two separate cross-sectional sets of self-reported data collected from samples that completed the translated scale.

### Measures

#### Demographic variables

Participants in the study were asked to provide basic demographic details such as their age, sex, and level of education.

#### MIAS scale

Afterward the participants were instructed to complete the MIAS, which encompasses 11 items. Respondents were asked to indicate level of agreement on a 5-point Likert scale, where 1 = strongly disagree, and 5 = strongly agree. The MIAS score varies from 11 to 55, where a higher score signifies higher stigma (17). Note that items 5, 6, 8, 9, 10, and 11 are reversed. Table 1 shows the MIAS items.

**Table 1:** The MIAS items.

Item 1 (St1)	I believe a person with mental illness is a danger to others
Item 2 (St2)	I believe a person with mental illness is unpredictable
Item 3 (St3)	I believe a person with mental illness is hard to talk with
Item 4 (St4)	I believe a person with mental illness has only himself/herself to blame for his/her condition
Item 5 (St5)	I believe a person with mental illness would improve if given treatment and support
Item 6 (St6)	I believe a person with mental illness feels the way we all do at times
Item 7 (St7)	I believe a person with mental illness could pull himself/herself together if he/she wanted
Item 8 (St8)	I believe a person with mental illness can eventually recover
Item 9 (St9)	I believe a person with mental illness can be as successful at work as others
Item 10 (St10)	Treatment can help people with mental illness lead normal lives
Item 11 (St11)	People are generally caring and sympathetic to people with mental illness

### Translation of the MIAS and scale adaptation

In compliance with the recommendations made by Sousa et al. (18) for culturally transferring healthcare research tools, we started the translation process with the forward-backward method, and subsequently had our preliminary draft reviewed and approved by a board of mental health and research professionals. The initial pre-final Arabic version was then test-piloted with a group of 10 individuals. Participants were instructed to review the scale's directions and elements utilizing a binary clarity assessment (clear or unclear). If any aspect of the tool was deemed unclear, feedback and suggestions for revisions were actively solicited from the participants to enhance clarity. Any component that was identified as unclear by a minimum of 20% of the sample required further scrutiny (18). Our results showed that all the 11 items reached a consensus level of 80% or more.

### Participants and data collection

#### Sample 1: test re-test reliability

The accepted norm for sample size in test-retest reliability studies, as evidenced by existing literature, suggests a participant count ranging from 20 to 40 (19, 20). In June 2023, an electronic questionnaire was presented to a randomly chosen group of 55 Arabic-speaking adults from the general population of Saudi Arabia. The ZDataCloud data collection system was employed to automatically determine eligibility (21, 22), which was based on being 18 years or older and using Arabic as a primary language. Qualified individuals from our participant database were notified via SMS to complete the survey through unique survey links. The decision to administer online SMS was driven by several factors. Online surveys provide accessibility and convenience, crucial for sensitive topics like attitude toward mental illness, ensuring higher participation rates and more honest responses. The anonymity of online responses is particularly vital in attitude toward mental illness research, as it encourages openness and honesty among participants, who might otherwise feel uncomfortable discussing such topics in person. Additionally, the online format allows for a wider demographic reach, essential for capturing diverse perspectives on attitude toward mental illness. This method also aligns with current social distancing norms, ensuring participant safety amidst ongoing health concerns. Up to three reminders were sent to each prospective participant within a 1 week period. It was imperative that participants fully complete all questions prior to submitting the questionnaire. The ZDataCloud system, equipped with integrated eligibility and sampling modules, was employed to maintain sample eligibility, manage distribution, avoid sampling bias linked to human error, and ensure data quality and integrity. Each response had to be fully answered for successful submission to the database. All gathered data were coded and securely housed within the ZDataCloud database.

#### Sample 2: structural validity

The suggested sample size for testing structural validity typically falls between a minimum of 2 and a maximum of 20 individuals per item. Given the presence of 11 items in the MIAS, the lower limit for our sample size was calculated to be 165 participants, based on the requirement of at least 15 participants per item (21, 23).

In June 2023, we selected a total of 300 Arabic-speaking adults from Saudi Arabia randomly to complete the digital questionnaire, considering potential non-responses. The completion of all questions was mandatory prior to submitting the questionnaire. The criteria for eligibility and the recruitment approach mirrored those implemented during the test-retest phase.

### Data analysis

We employed descriptive statistics to provide an overview of the sample and the corresponding MIAS scores. The internal consistency of the instrument was evaluated using Cronbach's α, while the test-retest reliability was gauged via the Intraclass Correlation Coefficient (ICC). The previously identified 2-factor structure from the original study was evaluated using Confirmatory factor analysis (CFA).

The appropriateness of conducting factor analysis was determined through an examination of the correlation amongst scale items, utilizing the Kaiser–Meyer–Olkin measure of sampling adequacy (with non-significant results indicating the data's suitability for factor analysis) and the Bartlett test (significant results indicating data appropriateness for factor analysis) (23, 24).

In order to scrutinize the factorial structure of the scales, an exploratory factor analysis was executed using the principal factor extraction technique. The oblimin rotation, principal axis extraction, and parallel analysis methods were employed to derive coherent factorial structures and facilitate a comparison with the original study.

## Results

### Study samples

#### Sample 1

Of the 38 participants in sample 1 (test-retest reliability), 19 (50.0%) were male and the mean age was 36.1 years (range 18–75). In the analysis of test-retest reliability, the ICC was α = 0.631.

#### Sample 2

The dataset comprises 189 subjects. The sample is relatively balanced, with 100 females (52.9%) and 89 males (47.1%). Respondents range in age from 18 to 70 years, with a mean age of 36.5 (SD = 13.1). Regarding the level of education, 59.8% hold a bachelor's degree or above and 40.2% have less than bachelor's degree. All participants completed the 11-item scale, thereby leaving no missing data. The overall scale consistency is good (Cronbach's alpha: 0.663). Table 2 showed the Social-Demographics of sample 2.

**Table 2:** Demographics of sample.

**Social-demographics**	***n* (%)**
**Sample 1**	
Age (Mean) (range 18–75)	36.1 years
**Gender**	
Male	19 (50.0%)
Female	19 (50.0%)
**Sample 2**	
Age (Mean) (range 18–75)	36.5 years
**Gender**	
Male	89 (47.1%)
Female	100 (52.9%)
**Education**	
Less than bachelor	76 (40.2%)
Bachelor and above	113 (59.8%)

#### Validation results

The original validation study (17) posited that the instrument could adhere to a two-factor or three-factor structure. However, they favored the two-factor structure. The authors of the original study reported that the two factors explain 32% of the common variance among items. The structure has a Cronbach's alpha of 0.69 for Factor 1 (labeled Negative Stereotypes) and 0.66 for Factor 2 (labeled Recovery and Outcomes).

A Confirmatory factor analysis (CFA) was performed following the original validation study with two factors. The CFA model does not show a good fit with CFI = 0.685, TLI = 0.583, RMSEA = 0.156. One item (item 7) in factor 1 has negative loading (Table 3). This suggests that the factor structure might differ or possibly be unidimensional.

**Table 3:** Confirmatory factor analysis (CFA) with a two-factor solution based on the original study.

**Factor**	**Indicator/Item**	**Estimate**	**SE**	***Z***	***P***	**Standard estimate**
Negative stereotypes	St1	0.749	0.1240	6.04	< 0.001	0.573
Negative stereotypes	St2	0.738	0.1159	6.37	< 0.001	0.573
Negative stereotypes	St3	0.842	0.1220	6.91	< 0.001	0.693
Negative stereotypes	St4	0.388	0.1075	3.61	< 0.001	0.350
Negative stereotypes	St7	−0.380	0.1309	−2.90	0.004	−0.276
Recovery and Outcomes	St5	0.815	0.0828	9.84	< 0.001	0.692
Recovery and Outcomes	St6	0.588	0.1075	5.47	< 0.001	0.420
Recovery and Outcomes	St8	0.993	0.0800	12.41	< 0.001	0.832
Recovery and Outcomes	St9	0.723	0.0918	7.88	< 0.001	0.576
Recovery and Outcomes	St10	0.898	0.0890	10.08	< 0.001	0.703

To identify a suitable dimensional structure in the data, exploratory factor analysis (EFA) was performed. All 11 items were included into EFA model. Sample size is sufficient for EFA based on Kaiser-Meyer-Olkin test (value 0.755). Bartlett's test of sphericity [χ^2^ (55) = 575, *p* < 0.001] is statistically significant, which further confirms that items correlate with each other to the sufficient degree for EFA to be performed.

**Figure 1:**
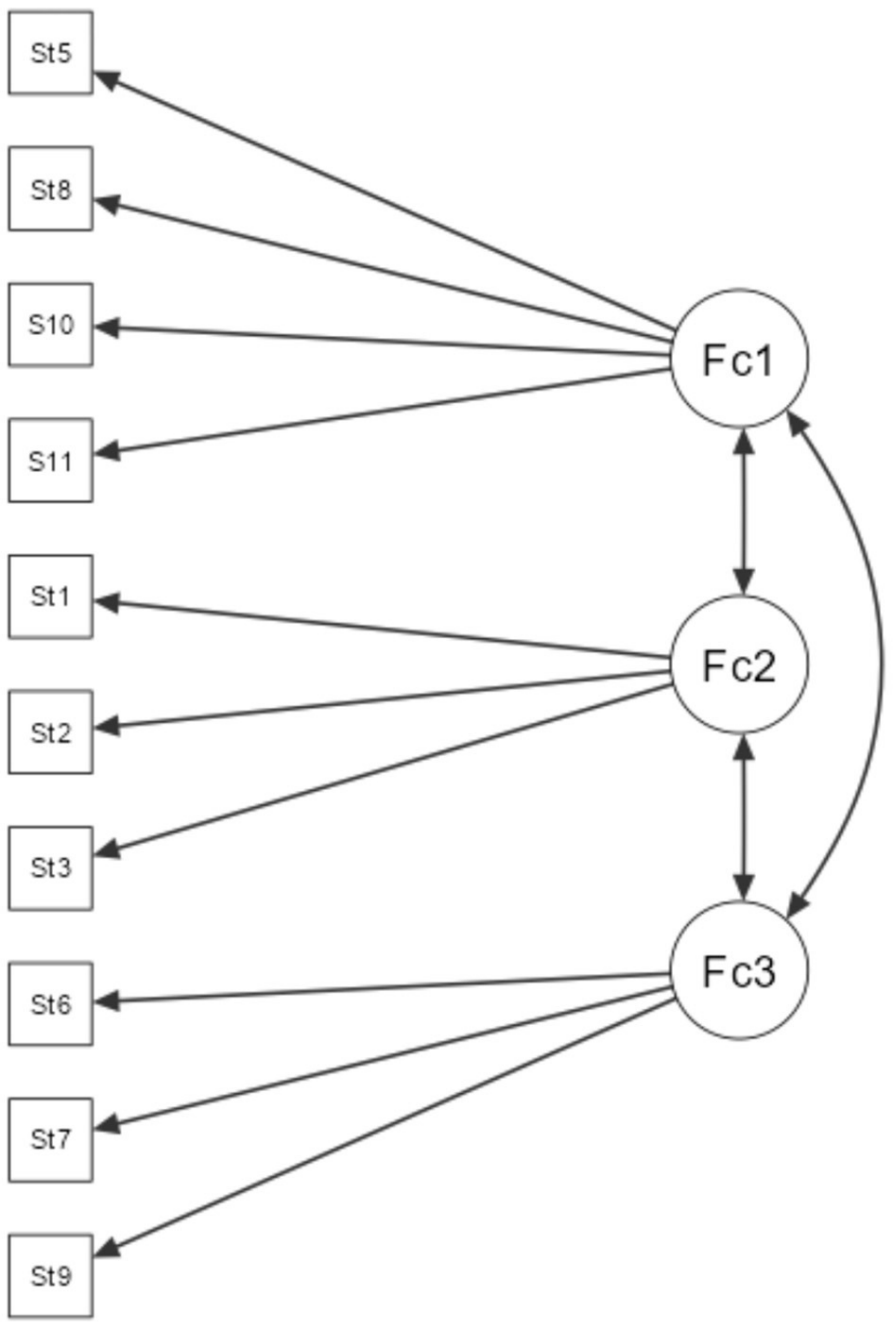
Exploratory factor analysis model.

The initial EFA model (oblimin rotation, principal axis extraction, parallel analysis) has 3 factors (Table 4). However, there is one item (item #4) that shows cross-loading between factor 1 and 2. Only the first two factors have eigenvalue > 1 (eigenvalue for factor 3 is 0.612). Three-factor model explains 47.2% of common variance and has a reasonably acceptable fit (TLI = 0.934, RMSEA = 0.057). Item 7 also have a negative loading in factor 3 (Figure 1).

**Table 4:** EFA results.

**Item**	**Factor**			**Uniqueness**
	**1**	**2**	**3**	
St1		0.520		0.578
St2		0.493		0.634
St3		0.755		0.435
St4	0.444	0.452		0.641
St5	0.684			0.490
St6			0.537	0.592
St7			−0.784	0.350
St8	0.731			0.372
St9			0.492	0.525
St10	0.785			0.397
St11	0.428			0.798

**Figure 2:**
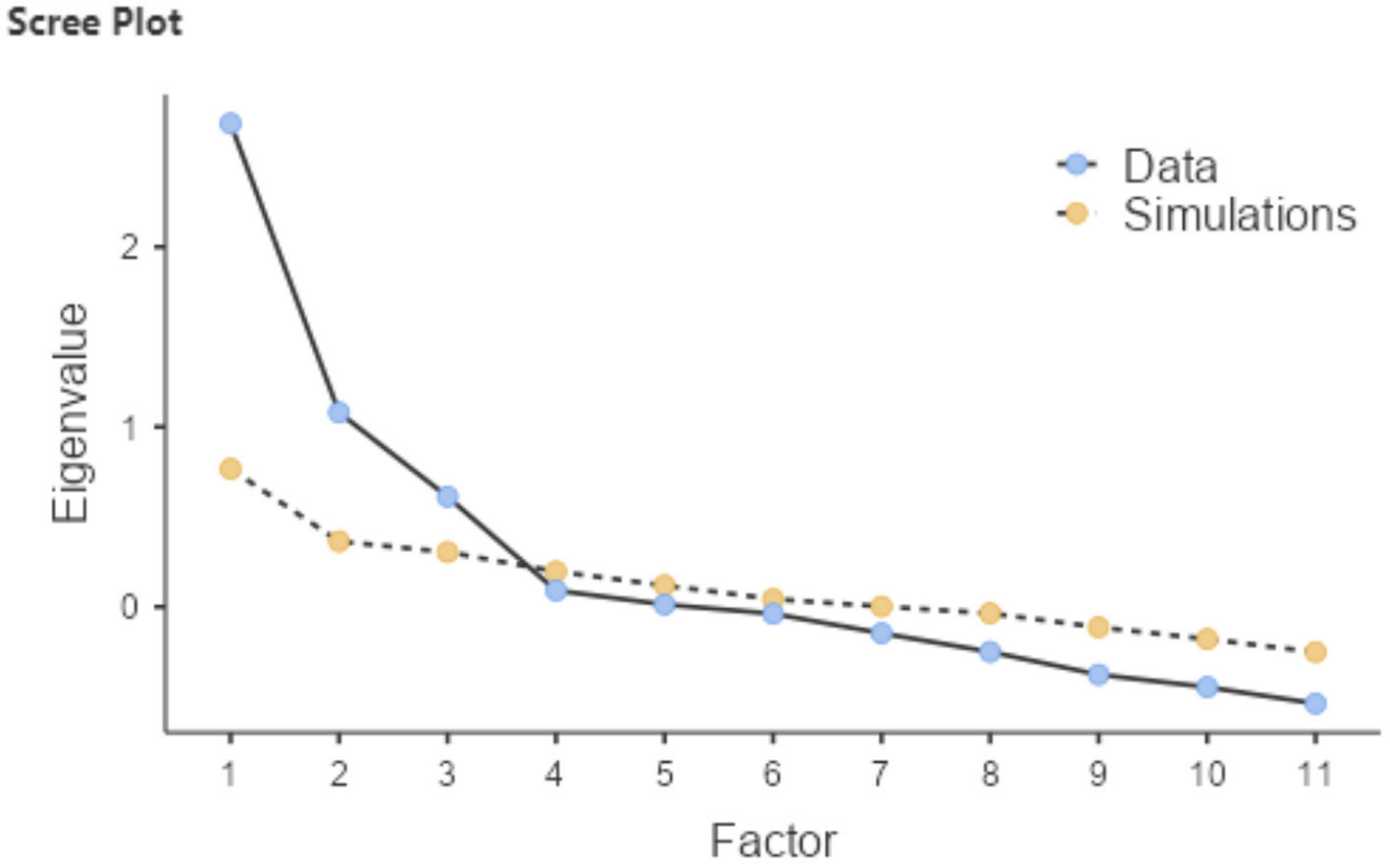
Scree plot.

According to Defne et al. (25), the optimal number of components for the model was determined using a scree plot (Figure 2), which suggested a clear ‘elbow' at the third component, indicating that additional factors contributed minimally to the explanation of variance within the data.

To determine three-factor model has a better fit, separate CFA model was built. Three-factor CFA model (Table 5) has a reasonably good fit (CFI = 0.890, TLI = 0.845, RMSEA = 0.094) and all items having significant loadings into the factors. Item 4 was excluded from the model as it does not fit with any of the three factors. Factor 1 includes 4 items (5, 8, 10, 11), has reliability of Cronbach's α = 0.77 and can be labeled as Outcomes. Factor 2 includes 3 items (1, 2, 3), has reliability of Cronbach's α = 0.65 and can be labeled as Negative Stereotypes. Factor 3 includes 3 items (6, 7, 9), but item 7 has negative coefficient. This is reflected with negative Cronbach's α = −0.87. Factor 3 can be labeled as Recovery. Significant strong positive correlation was found between factor 1 and factor 3, *r* = 0.659, *p* < 0.001. Factor 2 does not have significant correlation with factors 1 or 3. Nevertheless, it appears that three-factor model has a better fit and thus better reflects the dimensional structure of the instrument.

**Table 5:** Confirmatory factor analysis with 3 factors (following EFA).

**Factor**	**Indicator/Item**	**Estimate**	**SE**	***Z***	***p***	**Standard estimate**
Factor 1: Outcomes	St5	0.827	0.0819	10.09	< 0.001	0.702
Factor 1: Outcomes	St8	0.978	0.0797	12.27	< 0.001	0.819
Factor 1: Outcomes	St10	0.925	0.0884	10.46	< 0.001	0.724
Factor 1: Outcomes	St11	0.619	0.1017	6.09	< 0.001	0.462
Factor 2: Negative Stereotypes	St1	0.859	0.1490	5.76	< 0.001	0.657
Factor 2: Negative Stereotypes	St2	0.817	0.1404	5.82	< 0.001	0.635
Factor 2: Negative Stereotypes	St3	0.651	0.1156	5.63	< 0.001	0.536
Factor 3: Recovery	St6	0.757	0.1125	6.73	< 0.001	0.541
Factor 3: Recovery	St7	−0.898	0.1094	−8.21	< 0.001	−0.652
Factor 3: Recovery	St9	0.946	0.0965	9.81	< 0.001	0.754

## Discussion

### Results summary

This study focused on translating, validating, and psychometrically testing the Arabic version of the MIAS Scale within the Saudi Arabian general population. The Arabic MIAS exhibited good internal consistency, acceptable test-retest reliability, and produced a three-factor model. Each of the three factors showed good internal consistency. Our factor categorization is similar to that of the original study. However, unlike the original study which combined the “outcomes” and “recovery” factors into one, our study distinctly separates these two factors. Item 4 “I believe a person with mental illness has only himself/herself to blame for his/her condition” was excluded from the model since it didn't align with any of the three factors. Despite having a negative coefficient in the model, item 7 suggests a need for reverse coding. However, the logic behind the item implies that stigma increases if the participant strongly agrees with the statement: “I believe a person with mental illness could pull himself/herself together if he/she wanted.”

### Results interpretations

In terms of the internal consistency of the Arabic MIAS compared to the original English version the results are closely similar for of the sub-factors. It's noteworthy that the original MIAS scale study did not provide any test-retest reliability data or overall scale reliability which limited our comparison.

In considering the issues with items 4 and 7, it appears to be more a cultural and knowledge-based challenge in interpreting the meaning of the item, rather than a translation issue. Upon closer examination of item 4, the attribution of mental health conditions to the individual, and the view of such conditions as being subject to personal blame, can greatly vary across cultures, societies, and even within communities.

In the case of Saudi Arabia, which is largely a collectivist and spiritual society, several scientific studies exploring attitudes toward mental health have found these attitudes to be varied and complex. Some research does suggest that beliefs related to spiritual or supernatural causes, personal weakness, and divine punishment do exist within the society (26–28).

With the relatively low level of mental health awareness in Saudi Arabia, this interpretation is further complicated. For instance, one study found that 67.3% of participants believed depression was caused by a lack of faith, and 45.5% believed depression was caused by “the evil eye” or black magic. Consequently, this item could be interpreted bidirectionally, depending on individual beliefs, rather than as a general attitude (29).

A similar argument could be applied to item 7. Although this item fits within the model, it has been negatively interpreted by the participants. This item, too, pertains to the individual's responsibility for their mental health conditions. It appears that due to the cultural belief that an individual is solely responsible for their mental health condition, the item was logically linked to the “recovery” factor in the analysis, rather than the intended “Negative Stereotypes” factor, which also explains the negative coefficient.

This study is limited by several factors. First, there is a lack of other validated scales potentially usable for routine monitoring of stigma in population surveys. Second, due to the non-condition specific nature of this scale, no specifications regarding the type of mental illness were made (e.g., a person with dysthymia vs. a person with schizophrenia). As such, respondents were expected to self-define the construct of mental illness (30, 31). In theory, respondents' attitudes can vary based on their beliefs and feelings about the cause, nature, treatment, and prognosis of mental illness (32).

On the positive side, the translated scale demonstrated good validity. This contributes to the literature on non-condition-specific tools that can be used in assessing public attitudes toward mental health stigma, providing a broad understanding of societal attitudes and beliefs about mental illnesses. Further research is still needed to develop and improve such tools, enhancing their validity and cross-cultural applicability, to facilitate comparisons across countries and cultures.

## Conclusions

Our study supports the validity and reliability of the Arabic MIAS Scale among the Saudi population. However, cultural interpretation challenges related to personal blame and responsibility for mental health conditions emerged. The diversity of attitudes and beliefs about mental health in Saudi Arabia, compounded by low mental health awareness, further complexified the interpretation. Future research should focus on enhancing the validity of non-condition-specific tools and their cross-cultural applicability to advance understanding of mental health stigma across diverse contexts.

## Data availability statement

The raw data supporting the conclusions of this article will be made available by the authors, without undue reservation.

## Ethics statement

The studies involving humans were approved by the Ethics Committee of the Sharik Association for Health Research. The studies were conducted in accordance with the local legislation and institutional requirements. The participants provided their written informed consent to participate in this study.

## Author contributions

NB: Conceptualization, Data curation, Formal analysis, Investigation, Methodology, Software, Supervision, Validation, Writing – original draft, Writing – review & editing. NA: Data curation, Funding acquisition, Project administration, Supervision, Validation, Writing – review & editing. SA-L: Conceptualization, Writing – review & editing. MA: Conceptualization, Writing – review & editing. SS: Conceptualization, Writing – review & editing. HA: Conceptualization, Writing – review & editing. AS: Validation, Writing – review & editing. RA-D: Writing – review & editing. AA: Writing – review & editing.
